# Supplementary material for: Vitamin E-Loaded PLA- and PLGA-Based Core-Shell Nanoparticles: Synthesis, Structure Optimization and Controlled Drug Release
Source: Pharmaceutics. 2019 Jul 22;11(7):357. doi: 10.3390/pharmaceutics11070357 (PMC6680571; doi:10.3390/pharmaceutics11070357)
Supplement: Supplementary file 1 [file pharmaceutics-11-00357-s001.pdf]

pharmaceutics

Article

Supplementary Materials: Vitamin E-loaded PLA- and PLGA-Based Core-Shell Nanoparticles: Synthesis, Structure Optimization and Controlled Drug Release

Norbert Varga[1], Árpád Turcsányi[1], Viktória Hornok[1,2] and Edit Csapó[1,3]*

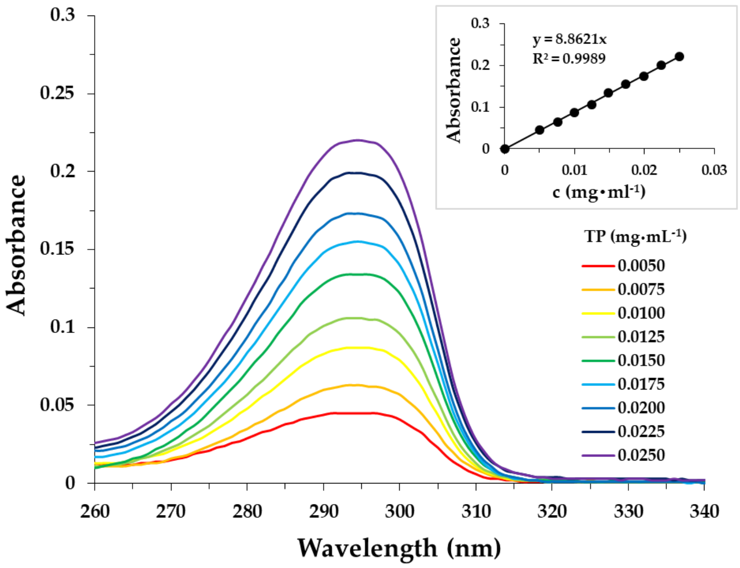

Figure S1. UV spectra of TP in 1,4-dioxane at different concentrations.

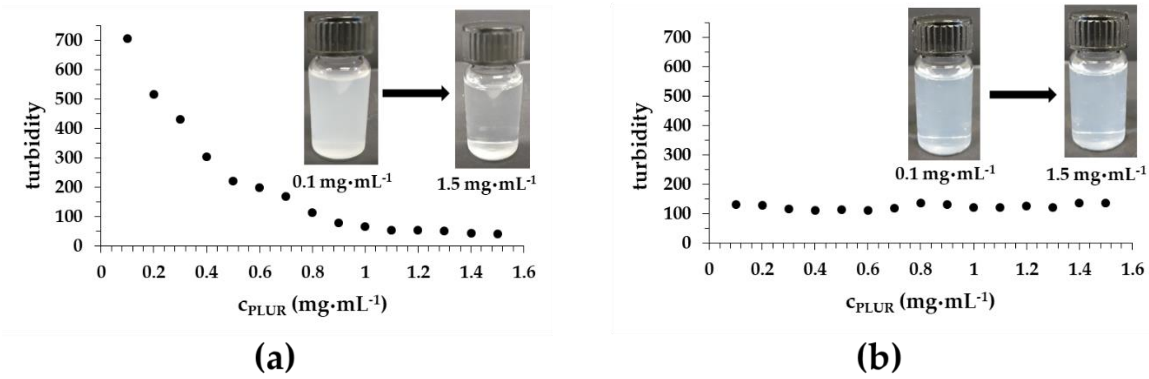

Figure S2. The turbidity of TP (a) and PLA (b) in PLUR solution at 25 °C in aqueous medium (c_{TP} = 0.25 mg•mL^{-1}, c_{PLA} = 0.25 mg•mL^{-1}).

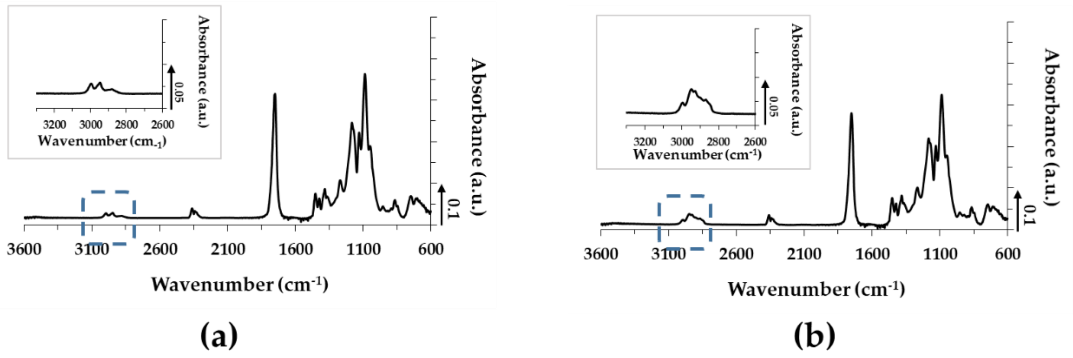

Figure S3. IR spectra of the TP-free (**a**) and TP-loaded (**b**) PLGA75 NPs (c_{TP} = 2.5 mg•mL^{-1}, c_{PLA} = 10 mg•mL^{-1}, c_{PLUR} = 0.1 mg•mL^{-1}).

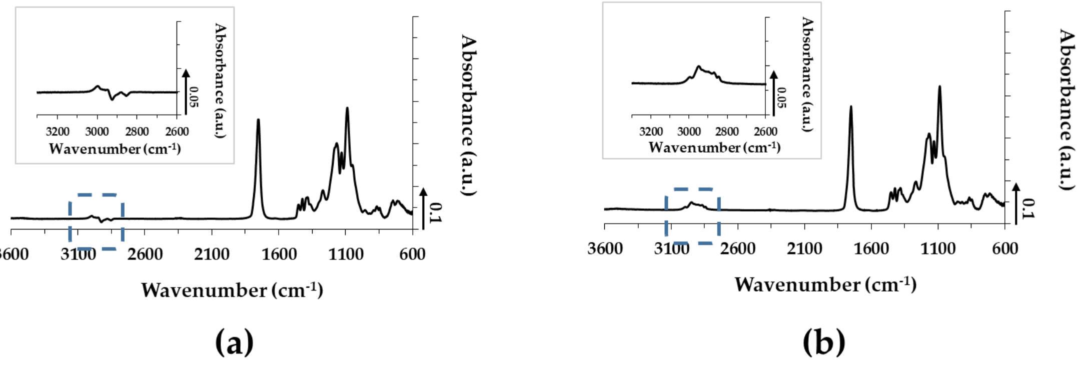

Figure S4. IR spectra of the TP-free (**a**) and TP-loaded (**b**) PLGA65 NPs (c_{TP} = 2.5 mg•mL^{-1}, c_{PLA} = 10 mg•mL^{-1}, c_{PLUR} = 0.1 mg•mL^{-1}).

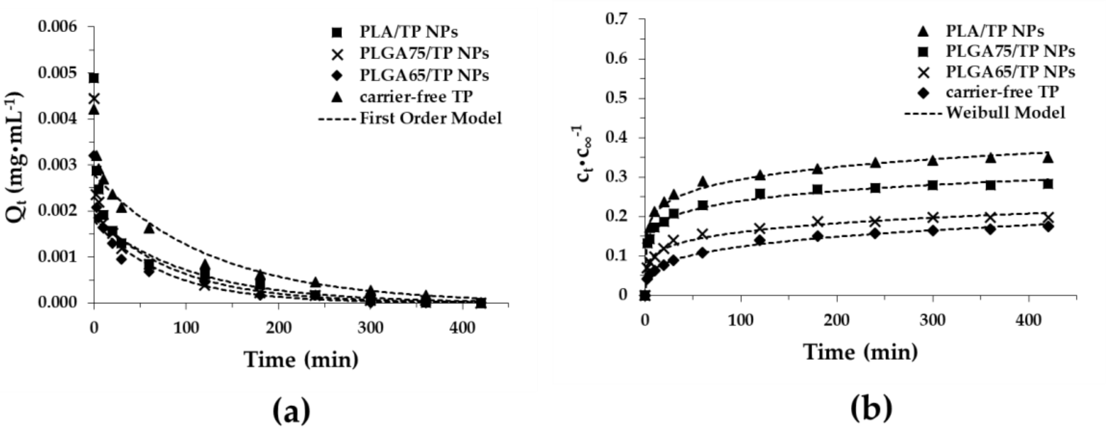

Figure S5. Release profiles and different kinetic models-predicted (First Order model (**a**), Weibull model (**b**)) release curves of TP from PLA and PLGA NPs in PBS solution (pH = 7.4, 0.9 w/w% NaCl).
